# Posterior bi-parietal decompressive craniectomy in refractory intracranial hypertension secondary to civilian gunshot wound. Case report and review of literature

**DOI:** 10.1016/j.ijscr.2018.10.045

**Published:** 2018-11-01

**Authors:** Guillermo Axayacalt Gutierrez-Aceves, Antonio Sosa-Najera, Alejandro Ceja-Espinosa, Jose Alfonso Franco Jimenez, Martinez-Maldonado Horus, Gabriel Ibarra-Trujillo, Carlos Tevera-Ovando, Diana Melani Saucillo-Lopez

**Affiliations:** a Radioneurosurgery Unit, National Institute of Neurology and Neurosurgery “Dr. Manuel Velasco Suarez”, México City, Mexico; b Neurological Center, Neurosurgery Department, American British Cowray Medical Center, México City, Mexico; c Neurosurgery Department, Centro Medico “Lic. Adolfo López Mateos”, Instituto de salud del estado de México, Av. Nicolas San Juan s/n Ex Hacienda la Magdalena, Estado de México, Mexico; d Médico Pasante del Servicio Social FES Iztacala, UNAM, Mexico

**Keywords:** Biparietal craniectomy, Traumatic brain injury, Intracranial hypertension

## Abstract

**Background:**

Decompressive craniectomy is recommended as second tier therapy for unresponsive intracranial hypertension in Traumatic Brain Injury. There have been reports of a Bi-Occipital craniectomy in cases where the focal injury is posterior.

**Case description:**

The work has been reported in line with the SCARE criteria. There is a 56-year-old male with Traumatic brain injury secondary to gunshot and intracranial hypertension, managed with biparietal craniectomy, after place a intracranial pressure monitor, whit good response to surgical and medical treatment, even with good outcome after hospitalization.

**Conclusions:**

In selected cases a posterior bi-parietal craniectomy can be performed in a safe way with acceptable results to treat refractory Intracranial hypertension. We propose that this neurosurgical technique can be used in patients with posterior focal injuries.

## Highlights

• Decompressive craniectomy is the therapy for unresponsive intracranial hypertension in Traumatic Brain Injury.
• There have been reports of a Bi-Occipital craniectomy in cases where the focal injury is posterior.
• Bi-parietal craniectomy can be performed in a safe way with acceptable results to treat refractory Intracranial hypertension.

## 1 Background

Traumatic Brain Injury [TBI] can cause intracranial hypertension refractory to medical treatment. Decompressive craniectomy is recommended as second tier therapy for unresponsive intracranial hypertension. Generally, large unilateral fronto-temporo-parietal decompression is used for focal injuries of one cerebral hemisphere, while bilateral frontal craniectomy is used for diffuse injuries [1]. There have been reports of a Bi-Occipital craniectomy in cases where the focal injury is greater in posterior cranial areas.^2^ We describe a case with refractory intracranial hypertension secondary to civilian gunshot wound, that was managed with a posterior bi-parietal decompressive craniectomy. This work has been reported in line with the SCARE criteria [3].

## 2 Case description

**Fig. 1:**
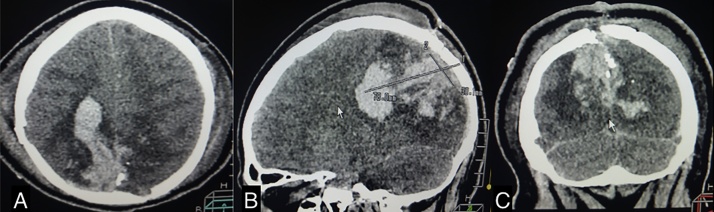
Axial, sagital and coronal non contrasted CT scan that shows intracerebral biparietal posterior haemorrhage.

We present the case of a 56-year-old male, admitted to the emergency room with [TBI] secondary to a gunshot wound in the right posterior parietal area. At admission he was awake with 11 points in the Glasgow Coma Scale [GCS] (O4 V2 M5), right pupil 4 mm, left pupil 3 mm, both light reactive. The initial CT scan (Fig. 1) showed the entry zone of the projectile almost in the midline, bi-parietal intracerebral hemorrhage that was predominant in the right side, with cerebral edema in both occipital lobes. Two hours after admission, he suffered neurological deterioration to 8 points in the GCS (O1 V2 M5) and required intensive care unit [ICU] medical treatment that include: intubation, mechanical ventilation, sedation and analgesia.

**Fig. 2:**
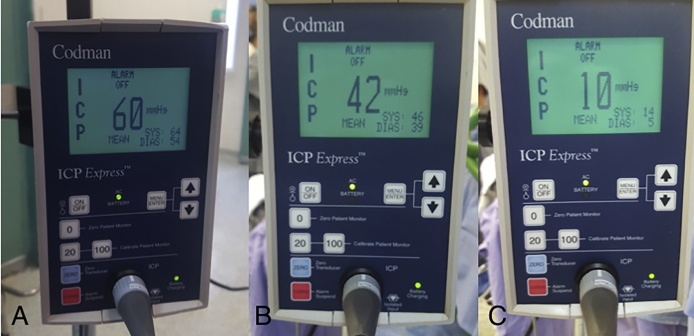
Codman ICP monitor showing pre, trans and postsurgical intracranial pressure.

We decided to place a parenchimal intracranial pressure monitor in the right Kocher point. The initial intracranial pressure was 60 mmHg, that was persistent despite optimal medical treatment. The patient was taken to the operating room and a posterior bi-parietal decompressive craniectomy was performed. After the Dural opening the intracranial pressure diminished until 42 mmHg; at the end of the surgery the intracranial pressure was 10 mmHg (Fig. 2).

**Fig. 3:**
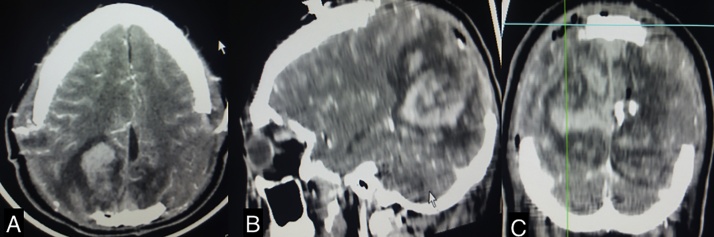
Axial, sagital and coronal postsurgical CT scan showing adequate cerebral decompression.

**Fig. 4:**
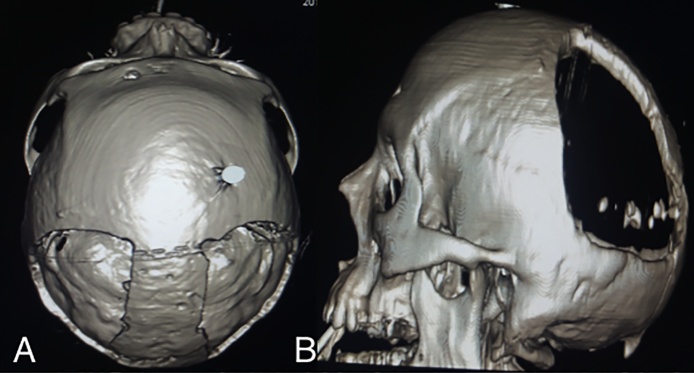
3D reconstruction showing postsurgical anatomy and extent of the posterior bi-parietal decompressive craniectomy.

Postoperatively the patient was admitted to the ICU where he was under intensive neuroprotective treatment with intracranial pressures under 15 mmHg. The 24 h postsurgical CT scan showed an adequate surgical decompression and presence of basal cisterns (Fig. 3, Fig. 4).

The patient was kept under sedation and analgesia with midazolam and propofol, maintaining a Richmond Analgesia-Sedation Score [RASS] -5, and the pupils returned to 2 mm, low reflective, he required vasopressor support (norepinephrine) to keep a mean arterial pressure between 90–100 mmHg. His diary urinary output was 1–1.2 ml/kg/h. At day 4 the sedatives were suspended to assess neurological response, however, six days after the surgery the patient underwent tracheostomy because the maximal Glasgow Coma Scale was 8 points; had diminished gag-reflex and developed nosocomial pneumonia. ICU discharged the patient to Neurosurgery service 12 days after, where he completed antibiotic treatment, started rehabilitation and physical therapy. At discharge of our service, at day 16 of hospitalization the patient was with 11 points in the GCS (O4, V1, M6), pupils were 3 mm both adequate light responsive, the surgical wound did not presented any complications, the strength in the 4 extremities was 4/5, reflexes ++, and sensibility was conserved in all of its modalities. Six months after the initial trauma the patient underwent cranioplasty with autologous bone graft.

## 3 Discussion

Trauma is the first cause of death in young population; especially patients under 40 years old and severe [TBI] contribute to a great number of these deaths. Currently there still are significant morbility and mortality associated to this type of patients and a low functional and vital outcome [4].

Refractory intracranial hypertension is a therapeutic challenge despite medical and neurosurgical advances, decompressive craniectomy [DC] is a second tier treatment, when optimal medical treatment is not enough to treat it; however despite is use, there are no specific studies about the effectiveness of this procedure according to each pathology [5].

During the last 30 years the medical and surgical therapies for the treatment of severe TBI have been deeply studied, that have made us understand the physiopathological features, and also the epidemiologic factors that influence the outcome of this patients such as age and trauma mechanism, even though it has been difficult to aim this knowledge to enhance the treatment of TBI patients through precise therapeutic guidelines [6].

Regard to the physiopathology of TBI, progress has been made in the comprehension of the mechanisms involved in cerebral swelling, intracranial hypertension, cerebral blood flow disturbances and neural metabolism [4].

After a TBI, the injured cerebral tissue swollens and because it is inside the closed cranial cavity the intracranial pressure rises, this can interfere with cerebral blood flow, causing ischemia and secondary damage to other areas not initially injured by the trauma [7].

Intracranial hypertension refractory to medical treatment in TBI has been treated with decompressive craniectomy as a second tier management, recent studies have shown evidence that DC can reduce intracranial pressure and have a favorable influence in outcome after a severe TBI [2,8].

There are also controversies in the type of DC (unilateral vs bilateral, anterior vs posterior, craniectomy limits, vascular tunnel formation to reduce venous congestion) [4]. In the case of focal injuries with midline shifting a unilateral DC is recommended, and in the case of diffuse injuries and cerebral swelling without midline shifting a bilateral DC is performed [8].

In most cases optimal medical treatment can control intracranial hypertension, this includes: supine position with cephalic elevation to 30 °, hypertonic solutions or manitol in bolus, hyperventilation for short periods and mild hypothermia, in case of persistence of IH, barbituric coma and CSF drainage are other therapeutic options. Factors of poor functional and vital outcome are: old age and low GCS at admission. It has been suggested that patients with brainstem disfunction should not be included for DC because of the poor outcome they have notwithstanding surgical treatment.

According to the 2012 Japan TBI guidelines, an IP monitor should be used in patients with GCS < 8, under sedation, and with an abnormal CT scan (Marshall II, III). The target values of IP are 15–20 mmHg to maintain a cerebral perfusion pressure between 60–70 mmHg. In cases that the IP is >30 mmHg despite optimal medical treatment it is recommended to perform an external surgical decompression (craniectomy) and/or internal (hematoma and contusions drainage, lobectomy, etc.) when the intraoperative IP maintains above 30 mmHg [9].

At present time the knowledge of cerebrovascular autorregulation, as the development of treatment guidelines, and scrutiny of conventional treatments (DC and IP monitoring) constitute a progress in patients with severe TBI, however, the advent of new medical, genetical and surgical strategies are the future preference of personalized treatments for each unique patient rather than following a “cook recipe” in TBI [7]. Sahuquillo et al. Reported in 2013 that a superior threshold in the IP has to be used in patients with refractory ICH, in order to select adequate candidates for surgical DC [11].

IP monitoring is a cornerstone in the treatment of patients with severe TBI, international guidelines indicate that there is still low amount of evidence to support level I or IIA recommendations, but when used mortality can be reduced [12].

In a randomized trial conducted by Chesnut et al in 2012, in South American hospitals, they compared the outcome between patients in two groups with severe TBI. The first group was treated based on continuous ICP monitoring and the second group was treated based on clinical/radiological findings. No statistical significative difference was found among the two groups in terms of cognitive and functional outcome. Mortality at 6 months in the ICP group was 39% vs 41% in clinical/radiological group, ICU length stay 12 days (ICPm) vs 9 days for clinical/radiological, duration of medical sedation 3.4 days (ICPm) vs 4.8 in clinical/radiological control group. Despite this results, they concluded that this trial was made in South American hospitals and some conditions differ from North American and European hospitals, so that more comparative studies have to be done in this matter [13].

There are not significative studies comparing DC against optical medical treatment in cranial gunshot wounds. There is no evidence to support DC as treatment of refractory IH, however some not randomized studies (ABTF guidelines) suggest a bifrontal DC within the first 48 h in the setting of diffuse swelling and IH refractory to optimal medical treatment [13].

The surgical decision can be difficult when contusions, swelling or intracerebral hemorrhage (ICH) are not “surgical” according to international guidelines, in this cases options are close clinical surveillance, CT scan if there is any clinical sign of neurological deterioration and ICPm. Some of the surgical recommendations include: GCS < 8 associated with diffuse injuries, neurological focal deficit, midline shift >5 mm, obliteration of basal cisterns, epidural hematoma >30 ml, subdural hematoma >10 mm in thickness or parenchimatous >50 ml [6].

In this case the decision to perform a posterior bi-parietal DC was made because of the focal injuries and hematomas in this area. It has been previously described that predominantly posterior injuries are feasible of treatment with a posterior DC [2]. One potential limitation of posterior DC compared with bi-frontal is the fact that the cerebral falx cannot be divided due to the risk of venous bleeding and secondary infarcts, that is why in this case we decided to maintain the midline bony fragment (and the second one was because the entry zone was in the midline so we decided to avoid manipulation of the venous sinus probably injured). There are some advantages of posterior DC, such as, no need to open frontal sinus, which carries a 2-0-8.5% risk of CSF fistula and cerebral infection, also as no bridging veins are present that cross the third posterior of the longitudinal sinus, once the dura is opened the brain can expand without traction to these veins. Finally, herniation of the brain through craniectomy is favored due to the gravitational force of the supine position in which the patient is maintained [2].

## 4 Conclusion

This case report shows that in selected cases a posterior bi-parietal craniectomy can be performed in a safe way with acceptable results to treat refractory IH. We propose that this neurosurgical technique can be used in patients with posterior focal injuries.

## Conflicts of interest

There is no financial or personal relationships with people or organizations that could influence this report. There is not a conflict of interest.

## Funding source

We do not have founding, the sources comes from us.

## Ethical approval

This study was exempted by ethical committee.

## Consent

The informed consent was obtained from the family and patient for publication.

## Author contribution

**Study conception and design:** Gutiérrez-Aceves G A and Sosa-Nájera A **Acquisition of data:** Gutiérrez-Aceves G A; Sosa-Nájera A and Ceja-Espinosa A **Analysis and interpretation of data:** Ceja-Espinosa A and Franco-Jiménez J A.

**Drafting of manuscript:** Gutiérrez-Aceves G A; Sosa-Nájera A, Ibarra-Trujillo G and Martínez- Maldonado H.

**Critical revision:** Telera-Ovando C and Saucillo-López D.

## Registration of research studies

NA.

## Guarantor

Antonio Sosa Nájera.

## References

[1] Hutchinson P.J., Kolias A.G., Timofeev I.S., Corteen E.A., Czosnyka M. (2016). Trial of Decompressive Craniectomy for Traumatic Intracranial Hypertension. N. Engl. J. Med..

[2] Stefini R., Bergomi R., Catenacci E., Cereda C., Latronico N., Mortini P. (2007). Bi-occipital Decompressive Craniectomy in Refractory Post Traumatic Intracranial Hypertension: First Report of One Case. Br. J. Neurosurg..

[3] Agha R.A., Fowler A.J., Saetta A., Barai I., Rajmohan S., Orgill D.P., for the SCARE Group (2016). The SCARE statement: consensus-based surgical case report guidelines. Int. J. Surg..

[4] Hutchinson P.J., Menon D.K., Kirkpatrick P.J. (2005). Decompressive Craniectomy in Traumatic Brain Injury—Time for randomised trials?. Achta Neurochir..

[5] Kim K.T., Park J.K., Kang S.G., Cho K.S., Yoo D.S. (2009). Comparison of the effect of decompressive craniectomy on different neurosurgical diseases. Acta Neurochir..

[6] Valadka A.B., Robertson C.S. (2007). Surgery of cerebral trauma and associated critical care. Neurosurgery.

[7] Coplin W.M., Cullen N.K., Policherla P.N., Vinas F.C., Wilseck J.M. (2001). Safety and Feasibility of Craniectomy with Duraplasty as the Initial Surgical Intervention for Severe Traumatic Brain Injury. J. Trauma Acute Care Surg..

[8] Bullock M. (2006). Ross. Surgical Management of Acute Subdural Haematomas. Neurosurgery.

[9] Abe t., Aruga T., Ogawa T., 2012, Japan Guidelines for the Management of Sever Head Injury 2nd edition. Neurol Med Chir. 52:1-30. 10.2176/nmc.52.1 22278024

[11] Sahuquillo J., Martínez-Ricarte F., Poca M.A. (2013). Decompressive Craniectomy in Traumatic Brain Injury after the DECRA trial. Where do we stand?. Curr. Opin. Crit. Care.

[12] Carney N. (2017). Guidelines for the management of severe traumatic brain injury. Neurosurgery.

[13] Chesnut R.M., Temkin N., Carney N., Dikmen S. (2012). A trial of intracranial pressure monitoring in traumatic brain injury. N. Eng. J. Med..

